# The signatures of Anthropocene defaunation: cascading effects of the seed dispersal collapse

**DOI:** 10.1038/srep24820

**Published:** 2016-04-19

**Authors:** Néstor Pérez-Méndez, Pedro Jordano, Cristina García, Alfredo Valido

**Affiliations:** 1 Department of Integrative Ecology, Estación Biológica de Doñana (EBD-CSIC), C/Americo Vespucio s/n, La Cartuja, 41092 Sevilla, Spain; 2 Plant Biology, Centro de Investigação em Biodiversidade e Recursos Geneticos (CIBIO/InBio), Campus Agrário de Vairão, Rua Padre Armando Quintas, Vairão. 4485-661, Porto, Portugal

## Abstract

Anthropogenic activity is driving population declines and extinctions of large-bodied, fruit-eating animals worldwide. Loss of these frugivores is expected to trigger negative cascading effects on plant populations if remnant species fail to replace the seed dispersal services provided by the extinct frugivores. A collapse of seed dispersal may not only affect plant demography (i.e., lack of recruitment), but should also supress gene flow via seed dispersal. Yet little empirical data still exist demonstrating the genetic consequences of defaunation for animal-dispersed plant species. Here, we first document a significant reduction of seed dispersal distances along a gradient of human-driven defaunation, with increasing loss of large- and medium-bodied frugivores. We then show that local plant neighbourhoods have higher genetic similarity, and smaller effective population sizes when large seed dispersers become extinct (i.e., only small frugivores remain) or are even partially downgraded (i.e., medium-sized frugivores providing less efficient seed dispersal). Our results demonstrate that preservation of large frugivores is crucial to maintain functional seed dispersal services and their associated genetic imprints, a central conservation target. Early signals of reduced dispersal distances that accompany the Anthropogenic defaunation forecast multiple, cascading effects on plant populations.

A myriad of vertebrate species have experienced population declines and eventual extinctions matching human expansion, i.e. the “Anthropocene defaunation”^1^,^2^. Large-bodied species have been especially hardly hit, and as a result, many disturbed ecosystems currently host only small- to medium-bodied species^3^,^4^. If the remnant, extant species fail to provide pivotal services formerly assisted by vanishing large vertebrates, human-driven defaunation may trigger negative cascading effects on ecosystem dynamics^5^,^6^,^7^,^8^,^9^.

Large frugivores provide essential seed dispersal services to the plants they feed upon by the large proportions of seeds they disperse over long distances^10^,^11^,^12^. Thus, extinction of these larger species is expected to limit the extent of gene flow and, by doing so, to impact the spatial distribution of plant genetic variation^13^,^14^. These effects are of paramount importance because the magnitude and distribution of the genetic variation within and among plant populations determine their evolutionary potential and the ability to cope with environmental disturbances^15^,^16^.

Here we focus on a compelling case study where human-driven defaunation led to a significant downsizing of seed dispersers. Downsizing results from non-random extinction of large-bodied species or from the loss of large-bodied individuals within populations^2^,^6^. We examine whether the downsizing of frugivorous lizards (*Gallotia*, Lacertidae) in the Canary Islands has progressively impaired their seed dispersal services. We further test if this process had a distinct signal on the fine-scale spatial genetic structure of *Neochamaelea pulverulenta* (Rutaceae), a plant species that relies exclusively on these lizards for dispersal^17^. Large-bodied *Gallotia* lizards were abundant in the past but the arrival of first settlers (~2500 BP; see Methods) unleashed a lizard defaunation process resulting in a marked, present-day gradient of seed disperser downsizing across islands^18^. Gran Canaria still preserves sizeable populations of a large-bodied species, *G. stehlini*, a setting close to the original situation; Tenerife hosts abundant populations of the medium-sized *G. galloti*; whereas La Gomera only has an abundant, small-sized lizard, *G. caesaris* (Fig. 1; see Methods). Despite the high specificity of the mutualistic interaction we address in this study, we aim to document effects of frugivore downsizing which are generally relevant for more diversified assemblages characterized by limited functional redundancy among the mutualistic frugivores^9^,^11^,^19^.

We hypothesized that the reduction in frugivore body size would result in reduced dispersal distances. We further expected that these reductions would strengthen the fine-scale spatial genetic structure of the plants and, consequently, reduce effective population sizes mirroring the downsizing pattern. Our study sheds light on the (mis)functioning of increasingly defaunated ecosystems, ratcheting downwards in ecological and genetic diversity through the extinction of ecological interactions^20^,^21^.

To assess the consequences of lizard downsizing, we examined the extent of contemporary seed-dispersal distances (i.e., the linear distance between a dispersed seed and its assigned maternal plant) and effective-dispersal distances (i.e., distances between established juveniles and saplings, and their inferred maternal plants). Descriptors of seed dispersal distances (median, maximum) and frequency of long distance dispersal events (LDD hereafter; events >30 m- 95^th^ percentile) decreased significantly with the reduction of lizard body size (*P* < 0.01 for all comparisons among islands; Table 1). Similarly, the frequency of immigration events (i.e., seeds not from maternal plants within the plots) decreased in parallel to the reductions of lizard body size across islands (Table 1). In contrast, the frequency of short-distance dispersal (SDD hereafter; events <5 m- 20^th^ percentile- a distance threshold three times the canopy size of adult plants) significantly increased with lizard downsizing. These among islands differences are not attributable to differences in the spatial distribution of either individual plants or seed sampling points among plots (Fig. S1). The longest dispersal distance events were detected in the population hosting the largest lizards (*G. stehlini*, Gran Canaria, Fig. 2; Table 1). In turn, the populations in Tenerife, and especially in La Gomera, showed an over-representation of SDD events assisted by the smaller-bodied *G. galloti* and *G. caesaris*, respectively, lacking LDD and immigration events in the latter (Fig. 2; Table 1). Furthermore, the effective dispersal distances also showed a similar trend to those of dispersed seeds (Fig. S2). These effects were apparent even though by incorporating sub-adults we are including dispersal events from an unknown number of reproductive episodes. Thus, these effective dispersal patterns (Fig. S2) are the result of a continuous history of seed dispersal and subsequent successful establishment in each population.

Differences among islands in the seed dispersal distances inferred from the 1-ha study plots are also generalizable up to the island scale, given the results from the additional replicated populations in each island (Table S1). Frequencies of SDD events were consistently and significantly higher in populations hosting small- (La Gomera) to medium-sized (Tenerife) lizards when compared with populations hosting large-sized lizards (Gran Canaria) (Table S1). Additionally, a previous demographic study encompassing 42 *N. pulverulenta* populations throughout the three islands^22^ showed significantly reduced seedling recruitment >1 m away from the maternal canopy, mirroring this lizard downsizing gradient.

Variation in seed-dispersal and effective-dispersal distance distributions likely reflect contrasted movement patterns associated with the larger body size of lizards in Gran Canaria relative to the Tenerife and La Gomera species. Body size has been highlighted as an important trait positively co-varying with lizard home range area^23^,^24^. In addition, a number of studies have shown that these differences in lizard home range sizes translate into variation of seed dispersal distances^25^,^26^,^27^. The documented collapse of seed- and effective-dispersal distances in La Gomera relates not only to the reduced home range of *G. caesaris*, but also to the mismatch between the oversized fruits (see Methods) and the lizards size (Fig. 1). Thus, only sporadic short-distance dispersal events still remain in this population, where the lizards just remove the fruit pulp and fail to ingest and disperse seeds.

Lost dispersal services in downsized populations may result in permanent shifts in dispersal patterns, with a legacy of persisting dispersal collapse. Previous studies^28^ have reported shorter distances of seedlings to maternal trees for two close related *Commiphora* species in Madagascar (where the species interact with small-sized frugivores) when compared to continental South Africa. In our study, the signal of seed dispersal collapse persisted to the established sub-adults, pointing to a long-lasting consequence of frugivore downsizing at the early demographic stages.

Estimates of plant genetic diversity in terms of expected and observed heterozygosity, effective number of alleles, and allelic richness were similarly high across studied plots regardless of the marked differences in the seed dispersal patterns (Table 1; see also Table S2 for results with sub-adult plants). Yet extensive pollen-mediated gene flow might counterbalance the effect of the limited seed dispersal.

The absence of inter-island differences in the amount of within-plot plant genetic diversity contrasted with differences in its spatial distribution. When comparing the covariation of the genetic similarity (*r*_*ij*_) with distance among pairs of adult plants, we detected significant values spanning longer distances in Tenerife and La Gomera (up to 25 m) than in Gran Canaria (up to 15 m) (Fig. 3). We obtained a similar pattern for the autocorrelograms derived from sub-adult individuals (saplings and juveniles) (Fig. S3). These trends were also confirmed by using Bayesian clustering analysis, where clusters represent groups of individuals in Hardy-Weinberg equilibrium (Fig. 3). We found an increasing number of genetic clusters from Gran Canaria (*K* = 1) to Tenerife (*K* = 2), and La Gomera (*K* = 3) mirroring the lizard downsizing gradient (Fig. 3).

Our results suggest a functional link between defaunation-driven downsizing, reduced seed- and effective-dispersal distances, and increased intra-population spatial genetic structure (SGS). These effects showed up at the scale of the 1 ha plots where the potential confounding effects of other factors (e.g., differences in population fragmentation among islands) would have a marginal influence. The study plots were embedded within large preserved populations that far exceed the home range sizes of *Gallotia* lizard species (see Methods). In addition, these plant populations present similar abiotic (temperature, rainfall regimes, soil type) and biotic characteristics (pollinator assemblages, vegetation composition, etc)^22^. Thus, we conclude that enviromental effects do not account for the differences observed among islands, according to the spatial scale of the study. Note that *Gallotia* lizards are the only seed dispersers of *N. pulverulenta*; thus, the observed reduction of seed dispersal distances following the downsizing is expected to leave a footprint in the spatial distribution of the genetic variation in the long term^29^,^30^. Estimates of effective population size (Ne/N) in the three situations confirmed this, with a significant reduction in the two islands with most downsized lizards (Tenerife and La Gomera; Table 1). The lowest value obtained in Tenerife is likely related to the fact that its demographic census (N) almost doubled the estimate for La Gomera, potentially influencing its smaller effective population size. Thus, defaunation may also impact the pollination stage by contributing to the emergence of local neighbourhoods with high genetic similarity. Mating events would preferentially occur among relatives within these small neighbourhoods^31^.

Our results support predictions of increased genetic structure when seed-mediated gene flow is reduced^13^,^28^,^32^,^33^. In addition, the results from Tenerife suggest that even moderate reductions in dispersal distances (i.e. medium sized lizards still providing sub-efficient seed dispersal) can impair the fine-scale genetic structure of *N. pulverulenta* populations. These findings warn of important alterations in the spatial distribution of plant genetic variation in systems apparently intact after frugivore defaunation, but with partially downgraded dispersal services.

In sum, the downgrading of seed dispersal services, following body-size reduction of mutualistic frugivores, cascades to the spatial genetic structure with potential negative consequences for plant populations in the long term. These effects include the collapse of the seed dispersal distances, which in turn enhanced fine-scale spatial genetic structure and reduced effective population sizes. We expect similar effects in more diversified frugivore assemblages where limited functional redundancy among species constrains possibilities for ecological replacement^11^,^19^. Preserving large frugivores is essential to maintain the functional seed dispersal services, their associated genetic imprints, and the potential for adaptation to environmental changes. Otherwise, the legacy effects of defaunation will eventually arise generations after the loss of important seed dispersers. Our study identified early signals of collapsed seed dispersal to forecast multiple, cascading effects on plant populations.

## Methods

### Study Species

*Neochamaelea pulverulenta* (Rutaceae) (Vent) Erdtman is a fleshy-fruited shrub (up to 2.7 m height), endemic to the Canary Islands (Gran Canaria, Tenerife and La Gomera). It is found in lowland xerophytic areas (Fig. S4), and is relatively abundant in some localities^34^. Ants and winged-insects (flies and bees) act as pollinators. Fleshy fruits include1–4 cocci, ripening from spring to early summer (Fig. S4). Each coccus can be considered functionally a drupe (11.1 ± 1.6 mm in diameter), containing one large, hard-coated seed (8.6 ± 1.0 mm in diameter)^35^. Only medium- to large-bodied Canarian endemic lizards (*Gallotia* spp., Lacertidae) consume these fruits and act as legitimate seed dispersers^17^,^22^,^35^,^36^.

**Figure 1:**
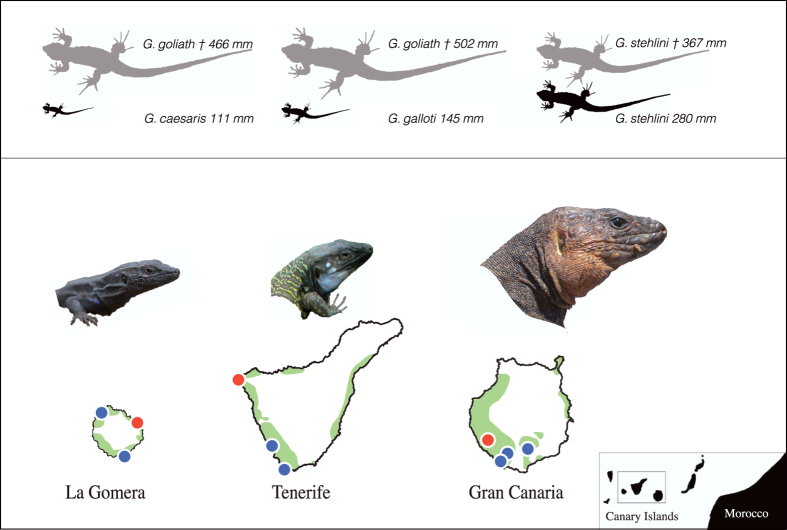
The extinction-driven lizard downsizing gradient in the Canary Islands. Schematic representation of the human-driven lizard defaunation resulting in present-day variable frugivore body-sizes among islands. *Neochamaelea pulverulenta* (Rutaceae) relies exclusively on the lizards for seed dispersal and is only found in lowland areas of Gran Canaria, Tenerife, and La Gomera (geographic range shown in green) (Fig. S4). Grey silhouettes illustrate extinct lizard taxa (†); black silhouettes represent the three extant, widely distributed species (photos). The maximum snout-to-vent length (SVL) is indicated. Red dots indicate locations of the main 1-ha study plots, whereas the blue ones indicate the replicated study populations (see Table S1). The potential geographic distribution of *N. pulverulenta* (green) was redrawn from ATLANTIS 3.1 (available on line, Banco de Datos de Biodiversidad de Canarias; http://www.biodiversidadcanarias.es/atlantis/). For details about natural history of these lizard species see also ref. 18 and 22.

Giant *Gallotia* lizard species were relatively abundant in the Canary Islands before the arrival of first settlers (c. 2490 ± 60 BP according to C_14_ dating)^18^,^37^,^38^. Nevertheless, the synergic combination of lizard hunting by humans and introduced mammals (e.g., feral cats), and a unique suite of life-history traits (e.g., low reproductive rate, absence of antipredatory strategies), most likely triggered the decline and/or extinction of these largest-sized species^18^,^39^,^40^. The magnitude of the extinction process and the resulting downsizing pattern notably varied among the three islands with *N. pulverulenta* populations^18^ (Fig. 1; see also Fig. S1 in ref. 22). For example, lizard downsizing has been relatively subtle in Gran Canaria, from the large individuals of *G. stehlini* sub-fossils (maximum snout-to-vent length; *max* SVL = 367 mm) to the extant individuals (*max* SVL = 280 mm). Intermediate downsizing has occurred in Tenerife, from the extinct giant *G. goliath* species (*max* SVL = 502 mm) to the medium-sized *G. galloti* (*max* SVL = 144 mm). In turn, a substantial body size reduction has been recorded in La Gomera, where the extinct *G. goliath* (*max* SVL = 466 mm) was four times larger than the extant *G. caesaris* (*max* SVL = 111 mm) (Fig. 1). Also, the critically endangered, *Gallotia intermedia* (*max* SVL = 174 mm) and *Gallotia bravoana* (*max* SVL = 212 mm) inhabit Tenerife and La Gomera, respectively, but they survive in extremely reduced, relict populations on very inaccessible cliffs^41^,^42^. This present-day biogeographic scenario allowed us to define a gradient of lizard downsizing, with three contrasted ecological settings illustrating the loss of large frugivores to a variable extent (Fig. 1). To assess the consequences of lizard downsizing we analysed the seed- and effective-dispersal distances in three representative 1-ha plots (one per island) illustrating the lizard body size gradient. We also studied the current fine-scale spatial pattern of plant genetic variation within those plots. To further test for differences among islands we also sampled two or three additional *N. pulverulenta* populations in each island (see below).

### Study sites and sampling design

We chose 10 plant populations encompassing the full geographic range of the plant to represent typical, well-preserved xerophytic scrublands comparable in biotic and abiotic conditions^22^ (Fig. 1, Table S1). In three of these populations we set a representative 1-ha plot for exhaustive sampling and intensive study located at: Gran Canaria (65 × 154 m; Barranco de Veneguera), Tenerife (80 × 125 m; Teno Bajo) and La Gomera (80 × 125 m; Punta Llana). The goal was to obtain genotypic data for all plants and extensive seed sampling to assess dispersal distances. The remaining populations received more restricted sampling to obtain replicated seed dispersal data (see below). *N. pulverulenta* plants and *Gallotia* lizards are relatively abundant in all the study sites. In order to increase the likelihood to infer the mother plant of dispersed seeds, the main plot size (1 ha) was established according to previous information on lizard movement patterns^43^. We georreferenced (Leica GPS 1200;±1 cm accuracy), tagged, measured (stem basal diameter, height, horizontal canopy projection area) and collected fresh leaves for all *N. pulverulenta* individual plants within the study plots. Individuals plants were classified as adult (both reproductive and non-reproductive adults), and sub-adult individuals (juveniles and saplings) according to previous information^22^. In addition, in order to increase the likelihood of determining the maternal source of dispersed seeds and determine to what extent the immigration events were mediated by long dispersal distances, all adult individuals located within a 12-m buffer area around the perimeter of the studied plots were also tagged, geo-referenced and sampled for leaves. Given previous evidences that legitimate dispersal does not occur in La Gomera^22^,^35^, these buffer areas were only sampled in Gran Canaria and Tenerife plots, where seeds are effectively dispersed by lizards (see below). All collected leaves were silica-dried and stored until lab processing. We sampled leaves from 675 individuals in Gran Canaria (adults = 409; juveniles = 102; saplings = 164), 987 in Tenerife (adults = 778; juveniles = 148; saplings = 61) and 509 in La Gomera plots (adults = 397; juveniles = 86; saplings = 26). Also, 229 and 525 adult plants were sampled from the buffer areas in the Gran Canaria and Tenerife plots, respectively (Fig. S5 for spatial distribution of plants).

The characterization of seed dispersal patterns of *N. pulverulenta* was performed by a regular sampling of lizard-mediated dispersed along 10-m wide transects on Gran Canaria (13 60-m transects), Tenerife (9 125-m transects), and La Gomera (10 80-m transects). Transects spanning the whole of the studied area. Along these transects we regularly established and geo-referenced sampling points every 5 m along the progression line. Around these points we established a circular sampling area of 1 m^2^ where we collected and silica-dried stored all fresh lizard droppings containing seeds of *N. pulverulenta*. In addition, these 10 m wide transects were carefully scanned by 2–3 people to increase the number of sampled seeds (Fig. S6 for spatial distribution of dispersed seeds and adult plants). Numbers of seed sampling points were *n* = 408, 612, and 414 for Gran Canaria, Tenerife and La Gomera plots, respectively. We found no evidence of legitimate, lizard-dispersed seeds (i.e., defecated seeds within lizard droppings) in La Gomera plot. The small-sized lizards in this population (*G. caesaris*) can not swallow *N. pulverulenta* fruits, but may sporadically disperse some seeds when they handle fruits while biting off the fleshy pulp^22^,^35^; therefore, we collected and geo-referenced all seeds manipulated by lizards found along linear transects within this plot. Overall, for the main 1-ha plots we obtained the genotypes for 326 endocarps in Gran Canaria, 305 in Tenerife, and 62 in La Gomera.

The exhaustive sampling required for the 1-ha plot maternity assignments of dispersed seeds limited logistically our ability to achieve a fully replicated sampling design within islands. We designed a complementary sampling protocol to allow comparisons of seed dispersal patterns among islands with adequate replication. We selected seven additional *N. pulverulenta* populations, 3 in Gran Canaria (2 in Barranco de Arguineguín, 1 in Barranco de Fataga), 2 in Tenerife (Malpaís de Punta de Rasca, La Caleta de Adeje), and 2 in La Gomera (Barranco del Medio, Taguluche) (see Fig. 1 for locations). In each population we set up 1–3 linear transects to scan for fresh lizard droppings containing *N. pulverulenta* seeds. For each replicated sampling areas of 1 m^2^ we collected all dispersed seeds and sampled leaves from all the adult plants present within a 5-m radius plot. We sampled 5–20 plots and 15–35 seeds per population (see Table S1 for details). We obtained the frequency of SDD events for each population by averaging the percentage of seeds whose assigned mother plant was found within the 5-m radius.

### Microsatellite genotyping

DNA was isolated from dried leaves of plants and from endocarps of dispersed seeds (the woody part of the seed, which is maternally originated tissue, surrounding the embryo)^44^. DNA extraction of leaves and endocarps was performed following protocols for this species^45^. Briefly, DNA extraction of leaves was performed with a modified CTAB extraction method. In the case of DNA extraction from endocarps, we carefully separated them from the embryos, before breaking it into small fragments with a pair of pliers. This woody tissue was immersed in liquid nitrogen, grinded in a ball-mill (Mixer Mill MM301, Retsch, Germany), and a Qiagen DNeasy Plant Mini Kit was used for DNA extraction. 5 μL of DNA extraction at different concentrations for leaves and endocarps (DNA: H_2_0; 2:13 and 3:2 respectively) were used for the polymerase chain reaction (PCR). Plants and endocarps were genotyped for 12 and 10 specific microsatellite markers, respectively, specially designed for parentage assignment of dispersed seeds, and analysis of spatial genetic structure^45^. Two markers were excluded for endocarps due to a high rate of amplification errors. Amplified fragments were analysed on an ABI 3130xl and the scoring was manually assessed using GeneMapper 4.0 (Applied Biosystems) and LIZ 500 size standard. The scoring was performed independently by two people and crosschecked. The identity probability (*i.e*. the average probability that two unrelated individuals share the same multilocus genotype) based on the set of polymorphic microsatellite markers ranged between 6 × 10^−10^ in Punta Llana and 6 × 10^−8^ in Teno.

### Maternity and parentage analyses, seed- and effective-dispersal distances, LDD, and immigration rate

As endocarps are tissues of maternal origin, the source plant for all dispersed seeds was assigned by a full matching of the multilocus genotypes of endocarps and any of the candidate mother plants. Because obtaining high-quality DNA from thick-coated endocarps recovered from faeces after some period in the ground, we needed a criterion allowing the inference of a matched genotype given a maximum number of mismatches among paired leaf-endocarp. To do that, we used the algorithm implemented in the *ALLELEMATCH* package^46^ in R 3.1.1^47^, which groups samples (plants and endocarps) sharing an unique genotype profile. This algorithm finds similarities between samples accounting for missing or mismatch data. Then it uses this information to apply a hierarchical clustering method to identify groups of similar genotypes (unique genotype profiles) at an optimal threshold of missing/mismatches (Fig. S7). Thus, seeds were assigned to a maternal shrub when both adult plants and seeds shared the same genotype profile. All analysed endocarps were assigned to a single adult individual, as multiple matches were not detected. Seeds with genotype profiles not matching any candidate mother plant were considered as immigrant seeds, i.e., dispersed from non-sampled plants growing in the surroundings of the study plots, as these are embedded in extensive continuous populations. Immigration rate is expected to be high in a plot surrounded by dense plant populations given that many more potential reproductive plants remain non-sampled. The buffer area of Teno exhibited a higher plant density than the Veneguera one, however immigration rate was significantly lower. This suggests that our assessments of the effects of lizard downsizing on immigrant events are conservative.

**Figure 2:**
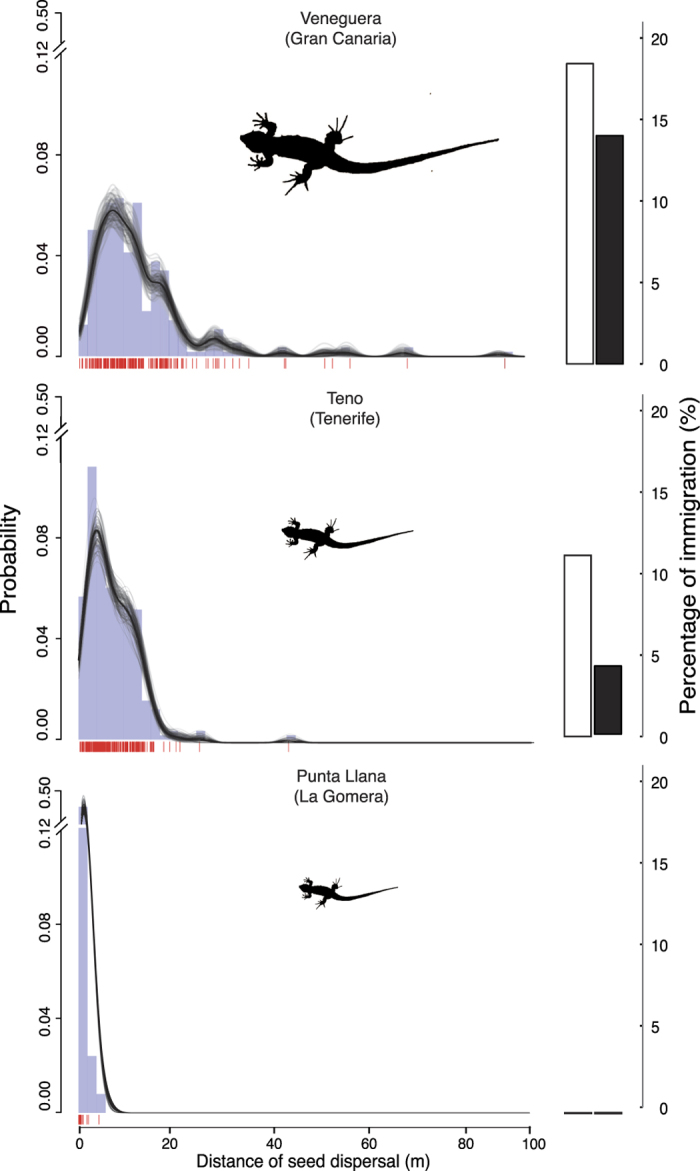
Seed dispersal patterns of *Neochamaelea pulverulenta*. Frequency distributions of seed dispersal distances (2-m bins). Vertical marks along the distance axis represent unique documented dispersal events. We included a non-parametric smoothing spline fit (black line) to the empirical distance distribution together with bootstrapped estimates (grey lines) to allow comparisons across plots. Right inset bars indicate the percentage of seed immigration from plants growing outside the plots (white) and when plants growing in the plot buffer area were also considered (black) (see Methods and Table 1 for sample sizes of dispersed seeds; maps shown in Figs S5 and S6).

Based on the spatial coordinates of both dispersed seeds and their assigned maternal plant we firstly measured the linear seed dispersal distances (±1 cm resolution). Secondly, we estimated the frequency distribution of dispersal distance events and the frequency of immigrant events. We tested for differences in the population-level, aggregated, frequency distributions of seed dispersal distances and immigration events resulting from multiple inferences of dispersal events sampled from dispersed seeds. To do that we resampled 999 times the empirical seed dispersal vectors (including immigrant seeds) to obtain bootstrapped estimates of dispersal distances and their associated SD. Then we applied linear models with island as fixed factor and a post-hoc Tukey test to evaluate among-island dispersal distance differences in terms of *i*) median, *ii*) maximum, but also *iii*) frequency of short distance dispersal (SDD; events <5 m), *iv*) frequency of long distance dispersal (LDD; events >30 m), *v*) frequency of immigrant events, and *vi*) frequency of immigrant events when accounting for plants growing in the12 m buffer. Finally, we adjusted a non-parametric function (smooth spline curve) and its bootstrap-estimated CI interval to the empirical frequency distribution of seed dispersal distances for each study plot to graphically represent the probability distribution of dispersal events (*n* = 99 resamplings; Fig. 2). Moreover in order to control for differences among plots in the aggregation and distribution of both reproductive plants and the sampling areas to collect dispersed seeds, we also calculated an expected distribution of seed dispersal distances. We estimated all the possible pairwise distances between reproductive plants and seed sampling points (Fig. S1). These expected distributions reflect the variable correspondence between the distribution of the plants and the sampling points^48^ due to simple positional effects. Among island differences in expected dispersal distributions (Fig. S1) are much subtler than differences between the expected and empirical seed dispersal distance distributions within islands. This suggests that differences among islands in the plants-dispersal sampling points arrangements (i.e., purely geometric effects) were not influential on the observed patterns.

The frequency distribution of effective dispersal distances were also estimated by applying parentage analyses to sub-adults individuals (saplings and juveniles separately) in 1-ha study plots. We used a maximum-likelihood framework as implemented in CERVUS 3.0 software^49^ to estimate both paternal and maternal parent for saplings and juveniles. Only reproductive individuals were considered as candidate parents. We used a relaxed 80% confidence interval for the parentage assignment and run 10,000 simulations with the following parameters: minimum number of matching loci = seven loci; error rate = 0.01; proportion of candidate parents sampled = 0.90; proportion of loci typed = 0.99. In addition, sample size (number of reproductive adults) and allele frequency were accommodated in the simulations for each study plot. As CERVUS does not differentiate between maternal and paternal parents, we assumed that the nearest reproductive individual was the maternal plant, whereas the most distant parent was the paternal plant. Our decision was based on information obtained in the Tenerife 1-ha plot (Teno) from a random subset of 40 dispersed seeds for which embryo genotypes were also obtained. Realized pollen dispersal distances, estimated from the embryo genotypes, were longer (median = 40.2 m) than their corresponding seed dispersal distances (3.2 m). Based on the spatial coordinates of sub-adult individuals and their inferred maternal plants, we also obtained the frequency distribution and the smoothing spline curve of effective dispersal distances of juvenile and sapling individuals in each studied plot (Fig. S2). The confidence intervals for the empirical seed dispersal distance distribution curves by bootstrapping were also estimated, as previously described.

### Genetic diversity, fine-scale spatial genetic structure and genetic clustering

We determined among-plot differences on the amount of plant genetic diversity in terms of expected heterozygosity (*uH*_*e*_), observed heterozygosity (*H*_*o*_), effective number of alleles (*A*_*e*_) and average allelic richness (*AR*) for each age class (i.e., adult, juvenile and sapling individuals) by applying GeneAlex 6.5^50^. In addition, we computed autocorrelograms for each plot and age class to compare the magnitude and spatial scale of the spatial genetic structure (SGS)^50^,^51^. This approach uses multilocus genotype information to calculate pairwise genetic autocorrelation coefficient (*r*_*ij*_) among pairs of individuals for a set of specified distance classes. Associated standard errors of *r*_*ij*_ were estimated using bootstrapping procedures. The selection of distance classes was calibrated to result in similar sample sizes. We used the same distance classes (5 m) in the three locations to allow comparisons among them. To test departures from the null-hypothesis (*i.e.*, for each distance class, two randomly chosen individuals are not more genetically similar than two individuals randomly chosen outside that given distance interval, *r*_*ij*_ = 0) we permuted the spatial position of individuals (*n* permutations = 999) and then calculated the mean of the 999 permuted values and generated a 95% confidence envelope around that null-hypothesis mean. Plant density is expected to enhance the spatial genetic structure (SGS) of plant populations, however our results showed that the studied plot in Tenerife (with medium-size lizards) showing the highest plant density exhibited lower SGS than La Gomera plot (small-sized lizards). Thus, variation in plant density seems not to obscure the signal imprinted by body-size reduction of lizards and the associated downgrading of dispersal functions. Finally, the effective population size (*Ne/N*) was calculated from genotype information of reproductive individuals by using the NeEstimator software^52^. We applied the linkage disequilibrium method excluding rare alleles occurring at lower frequencies than 0.01 (critical value).

We also explored fine-scale genetic structure within plots by searching for genetic clusters of adult plant. We first applied a Bayesian-clustering algorithm, as implemented in *GENELAND* R package^53^ that splits each plot into *K* clusters in Hardy-Weinberg-Linkage-Equilibrium (HWLE). We applied a two-step procedure^54^. Firstly, we performed 25 independent runs and checked for convergence issues after setting the following parameters: no admixture model; null allele model = TRUE; spatial model = TRUE; number max of nuclei = 778; uncertainty coordinates = 0; *K*_*min*_ = 1; *K*_*max*_ = 50 for 100000 sweeps; thinning = 100; burning = 100. Spatially-explicit models take into account the spatial location of the individuals to improve the inference power of the substructure when differentiation occurs by limited gene flow driven by the presence of physical barriers. The number of genetic clusters (*K*) was inferred as the modal number of genetic groups of the best run (based on posterior density values). Secondly, we fixed the number of genetic clusters inferred in the previous step for each study plot and performed 25 independent runs to assign each individual to a cluster. The assignment of each individual to a cluster was set according to the posterior probability of the cluster membership of the best run (threshold = 0.8). Then, we mapped the each individual sorted by clusters by using R 3.1.1^47^. When we inferred the genetic clustering for sub-adult individuals (sapling, juveniles), models did not converge properly, possibly because of low samples sizes. Thus, because the number of estimated clusters for each run varied considerably, we did not include the results for these age classes.

## Additional Information

**How to cite this article**: Pérez-Méndez, N. *et al*. The signatures of Anthropocene defaunation: cascading effects of the seed dispersal collapse. *Sci. Rep*. **6**, 24820; doi: 10.1038/srep24820 (2016).

## Supplementary Material

Supplementary Information

## Figures and Tables

**Figure 3:**
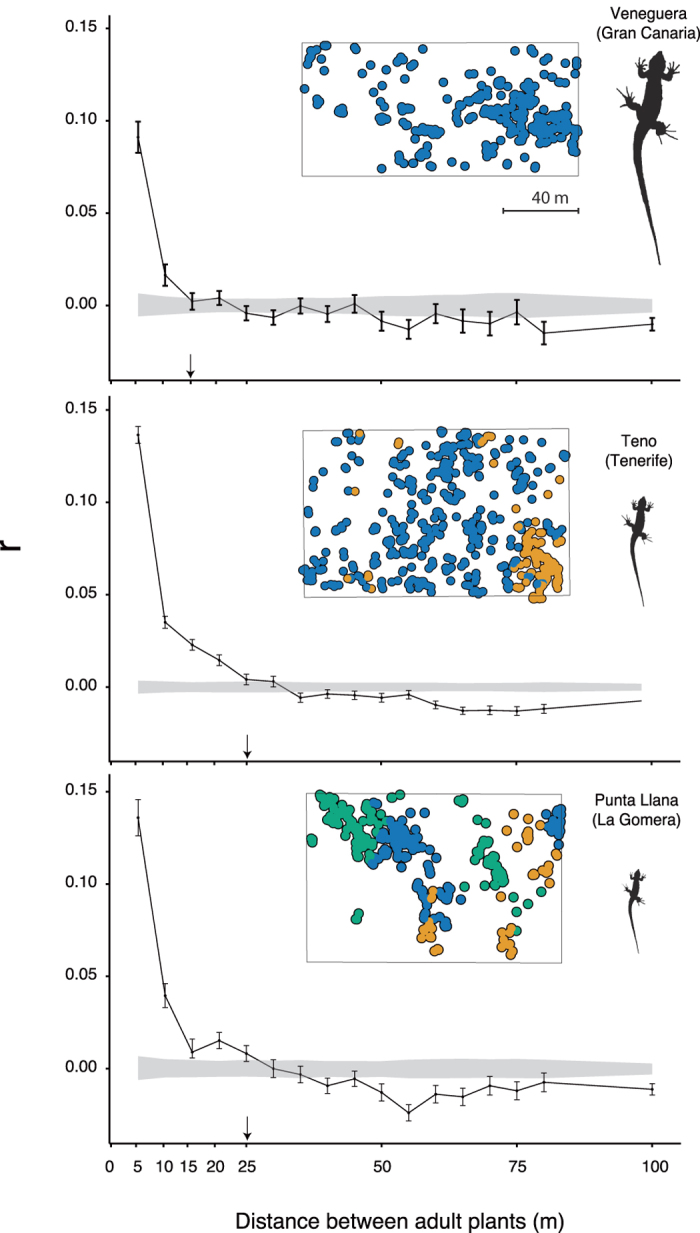
Spatial patterns of fine scale genetic structure of *Neochamelea pulverulenta*. Autocorrelograms showing the variation of genetic similarity (*r*_*ij*_) with geographic distance among pairs of adult plants (see Fig. S3 for results with sub-adults). Grey areas represent the 95% confidence intervals for the null hypothesis calculated by permutations of the plant spatial coordinates. Arrows indicate the first distance class at which the spatial autocorrelation becomes non-significant. Insets show the 1-ha plots maps indicating the distribution of adult plants (dots). Colours indicate the assignment of each plant to genetic clusters inferred according to the posterior probability of cluster membership (see Methods). All the study plots are represented at the same spatial scale.

**Table 1:** Summary of seed dispersal distance parameters (obtained from Fig. 2) and genetic diversity indices of *Neochamaelea pulverulenta* across the three 1-ha plots.

Population parameters	Gran Canaria(Veneguera)	Tenerife(Teno)	La Gomera(Punta Llana)
Median seed dispersal distance (m)	10.8 (10.7 ± 0.8)^a^	6.4 (6.2 ± 0.5)^b^	0.5 (0.5 ± 0.05)^c^
Maximum seed dispersal distance (m)	94.2 (84.3 ± 12.1)^a^	46.4 (37.1 ± 10.2)^b^	4.5 (3.6 ± 1.2)^c^
% dispersal events <5m (SDD)	17.2 (17.2 ± 2.5)^a^	41.6 (41.3 ± 2.8)^b^	100 (100 ± 0.0)^c^
% dispersal events > 30 m (LDD)	7.1 (7.4 ± 1.6)^a^	0.3 (0.4 ± 0.4)^b^	0 (0 ± 0.0)^c^
% immigration (plot)	19.1 (19.2 ± 2.3)^a^	11.4 (11.4 ± 1.7)^b^	0 (0 ± 0.0)^c^
% immigration (buffer)	14.7 (14.7 ± 2.1)^a^	4.6 (5.0 ± 1.2)^b^	0 (0 ± 0.0)^c^
Expected Heterozygosity (*uHe*)	0.64 ± 0.06	0.62 ± 0.06	0.64 ± 0.06
Observed Heterozygosity (*Ho*)	0.59 ± 0.06	0.60 ± 0.05	0.61 ± 0.06
Effective number of alleles (*Ae*)	3.5 ± 0.49	3.4 ± 0.54	4.0 ± 0.8
Allelic Richness (*AR*)	10.2 ± 1.6	10.2 ± 1.0	10.8 ± 1.4
Effective population size (*Ne/N*)	0.38 (0.35–0.42)	0.20 (0.18–0.22)	0.29 (0.27–0.32)

Sample sizes (dispersed seeds, adult plants) for seed dispersal distances parameters and genetic diversity indices for each plot are: Gran Canaria (326, 409), Tenerife (305, 778), and La Gomera (62, 397). Empirical values for dispersal distance parameters, % LDD, % SDD, and % immigration are indicated, and the bootstrapped estimates (and associated *SD*) are shown within brackets (different letters in superscripts indicate significant differences; see Methods). Genetic diversity indices are means ± 1 *SE*, and mean (95% *CI*) for effective population sizes.
