# Supplementary material for: The signatures of Anthropocene defaunation: cascading effects of the seed dispersal collapse
Source: Sci Rep. 2016 Apr 19;6:24820. doi: 10.1038/srep24820 (PMC4835773; doi:10.1038/srep24820)
Supplement: Supplementary Information [file srep24820-s1.pdf]

Supplementary Information for

The signatures of Anthropocene defaunation: cascading effects of the seed dispersal collapse

Néstor Pérez-Méndez[a], Pedro Jordano[a], Cristina García[b], and Alfredo Valido[a,*]

[a] Department of Integrative Ecology, Estación Biológica de Doñana (EBD-CSIC), C/Americo Vespucio s/n, La Cartuja, 41092 Sevilla, Spain; and [b] Plant Biology, Centro de Investigação em Biodiversidade e Recursos Geneticos (CIBIO/InBio), Campus Agrário de Vairão, Rua Padre Armando Quintas, Vairão. 4485-661, Porto, Portugal

[*]To whom correspondence should be addressed. Email: avalido@ebd.csic.es

Integrative Ecology Group, Estación Biológica de Doñana (EBD-CSIC), C/Americo Vespucio s/n, La Cartuja, 41092 Sevilla, Spain; Tel.: +34 954466700; fax: +34 954621125

Fig. S1 Frequency distributions of expected (i.e., distances among all pairwise reproductive *Neochamaelea pulverulenta* adult plants within plots to all the seed sampling points; grey bars, 5 m binning) and empirical, lizard-mediated, seed dispersal distances (black bars) in the three representative study 1-ha plots. Note the similarity of expected distances distributions among plots. Lizard silhouettes are scaled to the maximum SVL.

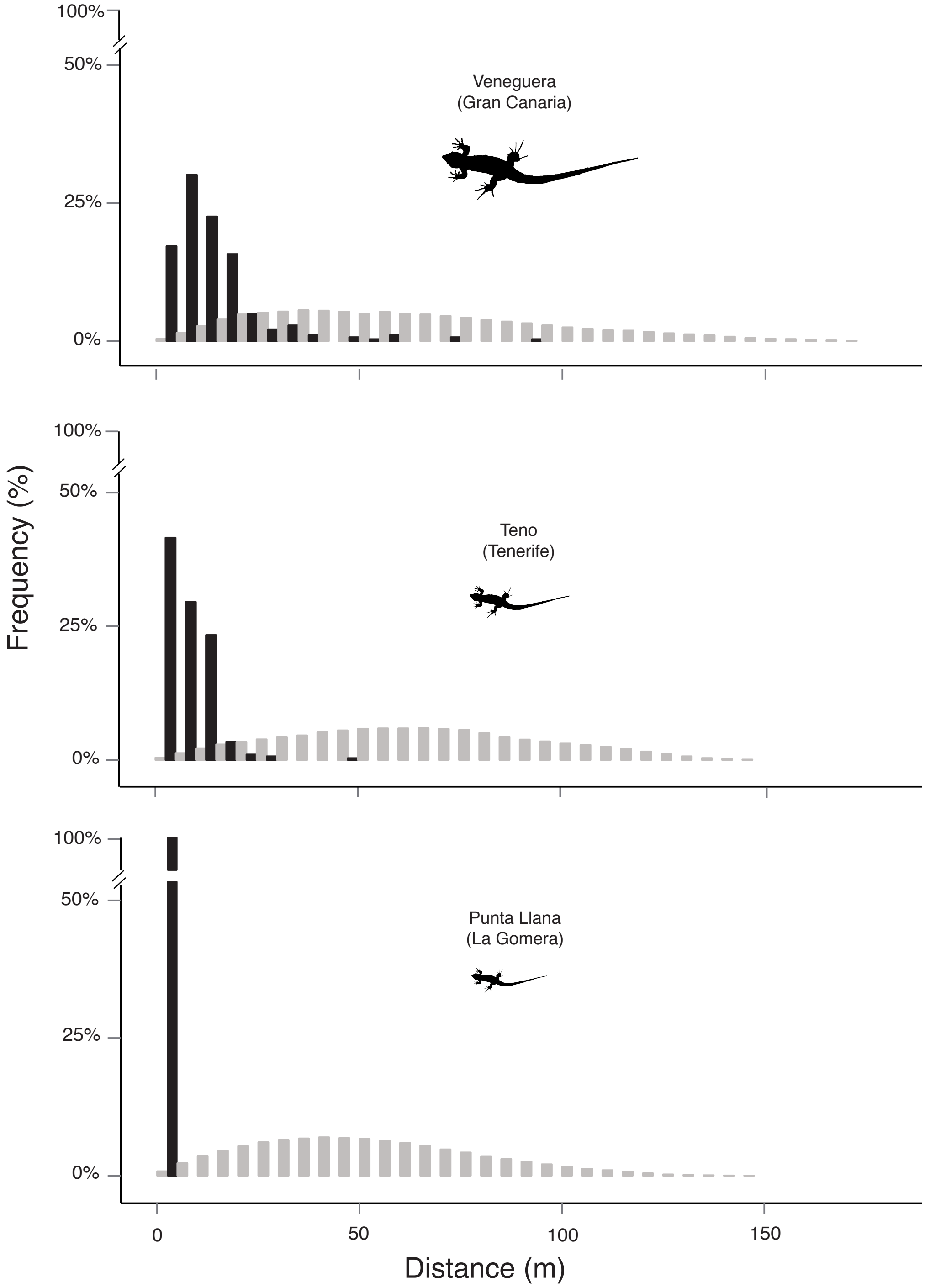

Fig. S2 Frequency distribution and smoothing curve of effective dispersal (i.e., the frequency distribution of distances between established saplings and juveniles and their assigned most likely maternal plant) of *Neochamaelea pulverulenta* in the three study 1-ha plots based on parentage analyses. Sample sizes (number of assigned recruits) are indicated in each panel. Maximum dispersal distances are: Veneguera (juveniles= 94.8 m; saplings= 81.9 m), Teno (juveniles= 55.4 m; saplings= 55.4 m) and Punta Llana (juveniles= 64.4 m; saplings= 38.7 m). We assumed that the maternal plant was the nearest candidate parent based on data about pollen and seed dispersal distances obtained from Teno (Tenerife) (see Methods). Lizard silhouettes are scaled to the maximum SVL.

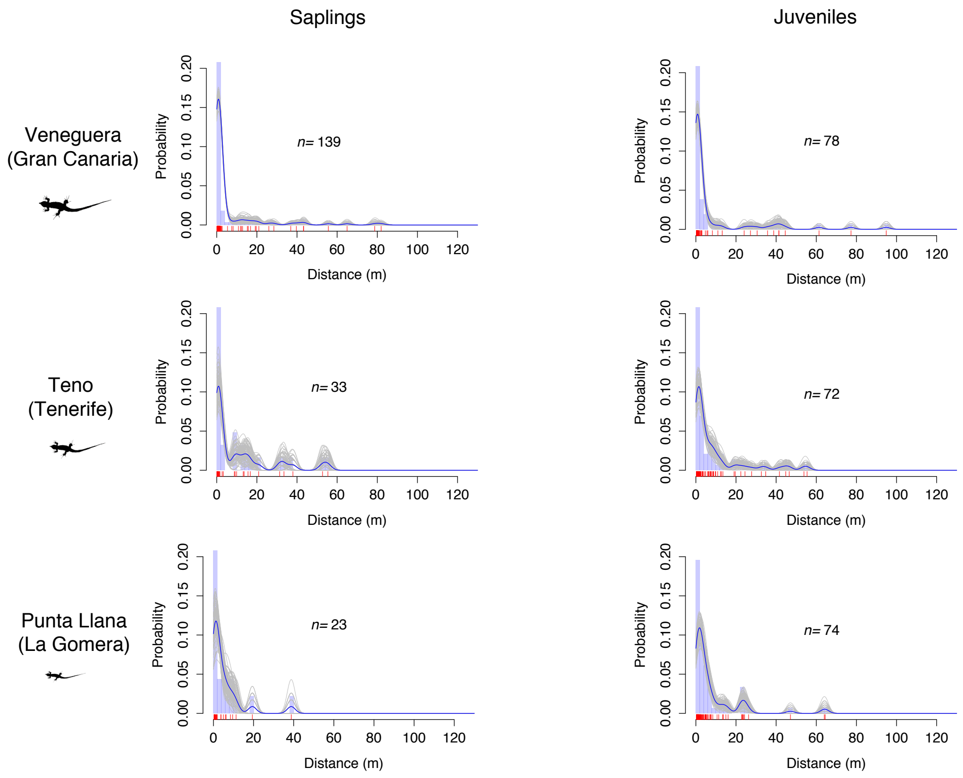

Fig. S3 Autocorrelogram of genetic similarity among pairs of *Neochamaelea pulverulenta* individual plants across the three 1-ha plots: Veneguera (Gran Canaria), Teno (Tenerife), and Punta Llana (La Gomera). Genetic similarity (r_{ij}) and error bars were determined by bootstrap resampling (n= 999) [51]. Grey areas represent the 95% confidence intervals for the null hypothesis calculated by permutations of the plant spatial coordinates. Distance classes are 1 m, 2 m, and successive 5 m intervals up to 80 m, and 100 m. Only plants growing within the study 1-ha plots were considered in this analysis. Lizard silhouettes are scaled to the maximum SVL.

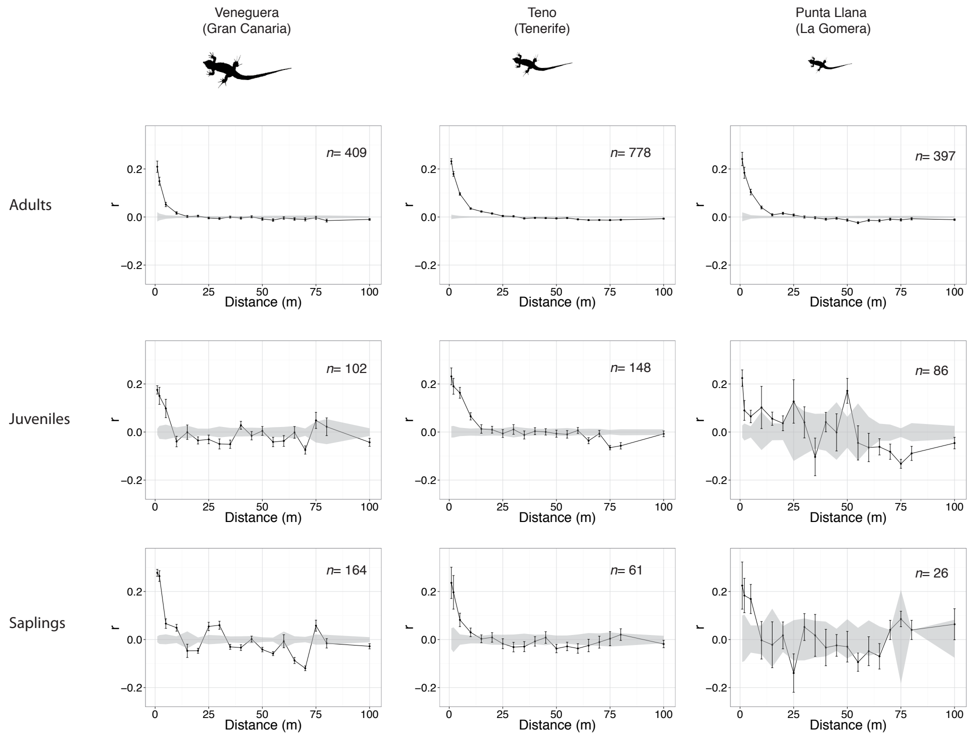

Fig. S4 Photo-collage shows: upper-left, *N. pulverulenta* individual growing close to an *Euphorbia balsamifera* and *E. canariensis* (Euphorbiaceae); upper-center, *N. pulverulenta* with ripe fruits; upper-right, details of a fruit formed by three cocci (functionally drupes); lower-left, study plots in Punta Llana (La Gomera); lower-center, Teno (Tenerife); lower-right, Veneguera (Gran Canaria).

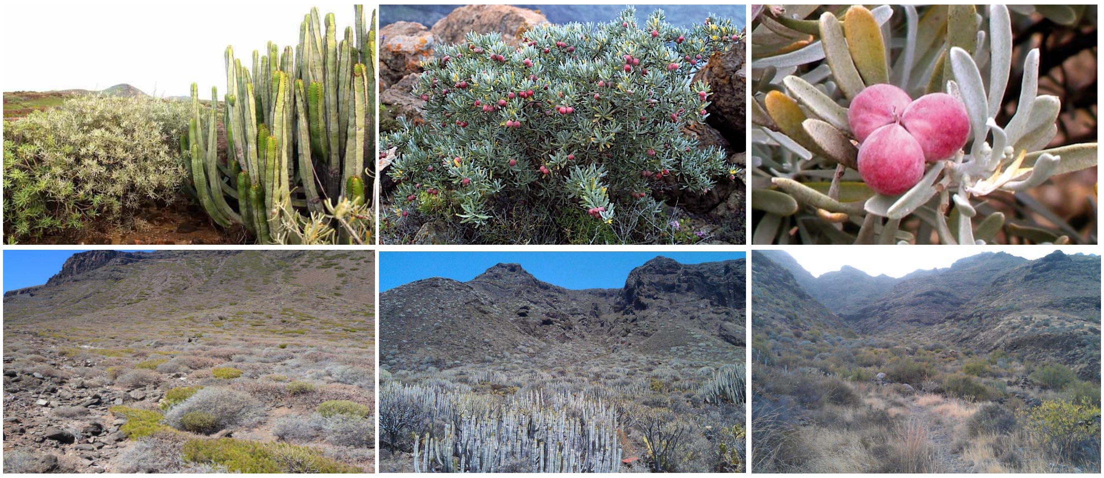

Fig. S5 Spatial distribution of adult (green circles), juvenile (white squares) and sapling (yellow triangles) individuals of *Neochamaelea pulverulenta* in the three study 1-ha plots: Veneguera (Gran Canaria; inhabited by *Gallotia stehlini* lizards), Teno (Tenerife; *G. galloti*), and Punta Llana (La Gomera; *G. caesaris*). The adult individuals sampled in the buffer area were also included as potential maternal plants for dispersed seeds sampled within the plot (*n*= 229 and 525 adults in Veneguera and Teno, respectively). The 1-ha plots are represented with continuous lines and the buffer limits with dashed lines. Veneguera (409; 102; 164, respectively), Teno (778; 148; 61, respectively), and Punta Llana (397; 86; 26, respectively). Lizard silhouettes are scaled to the maximum SVL.

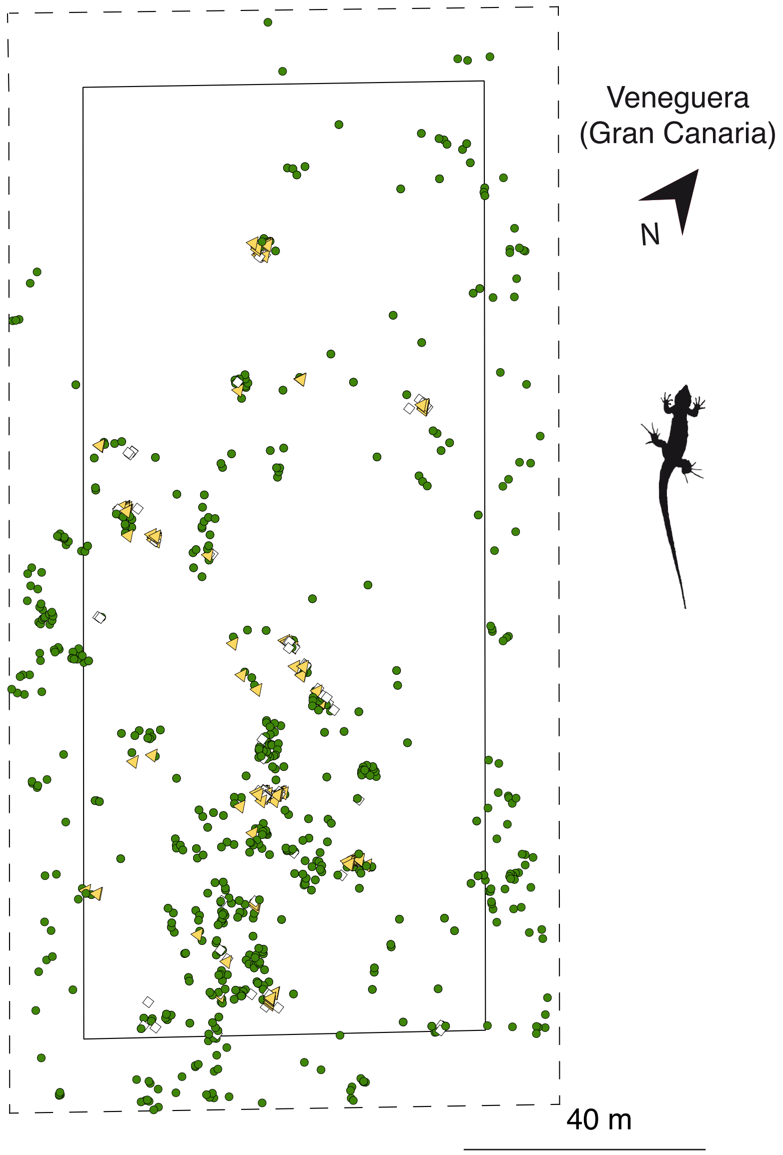

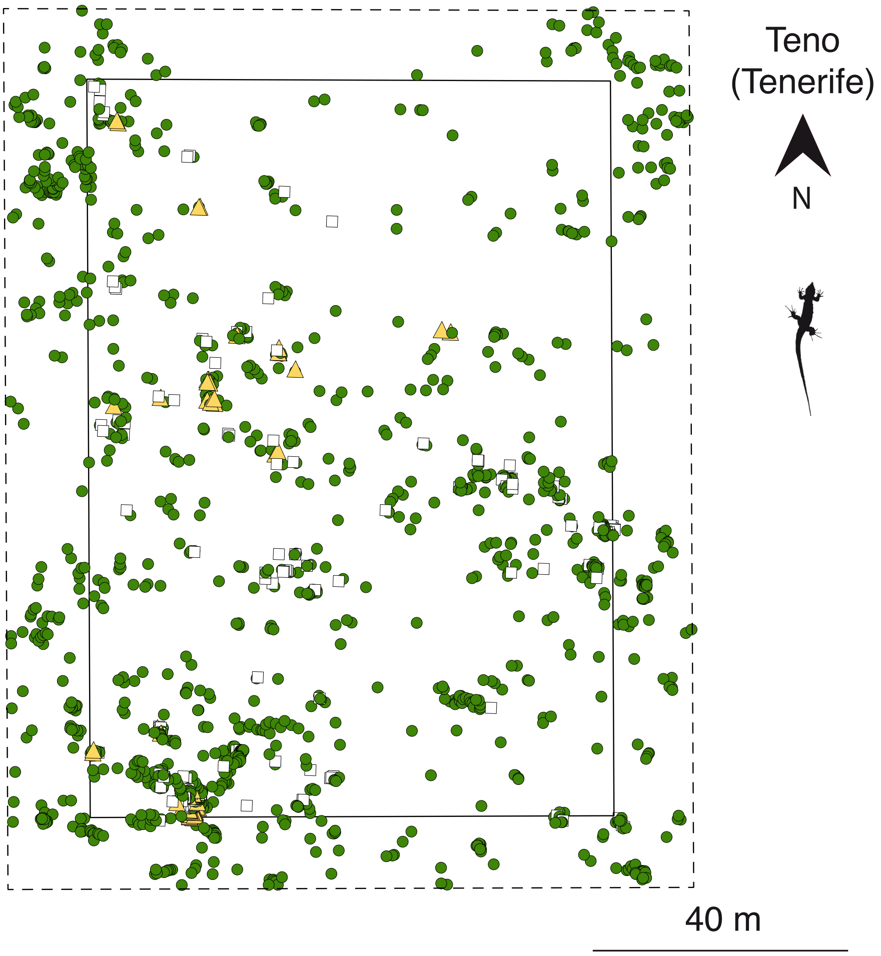

Teno
(Tenerife)
N
40 m

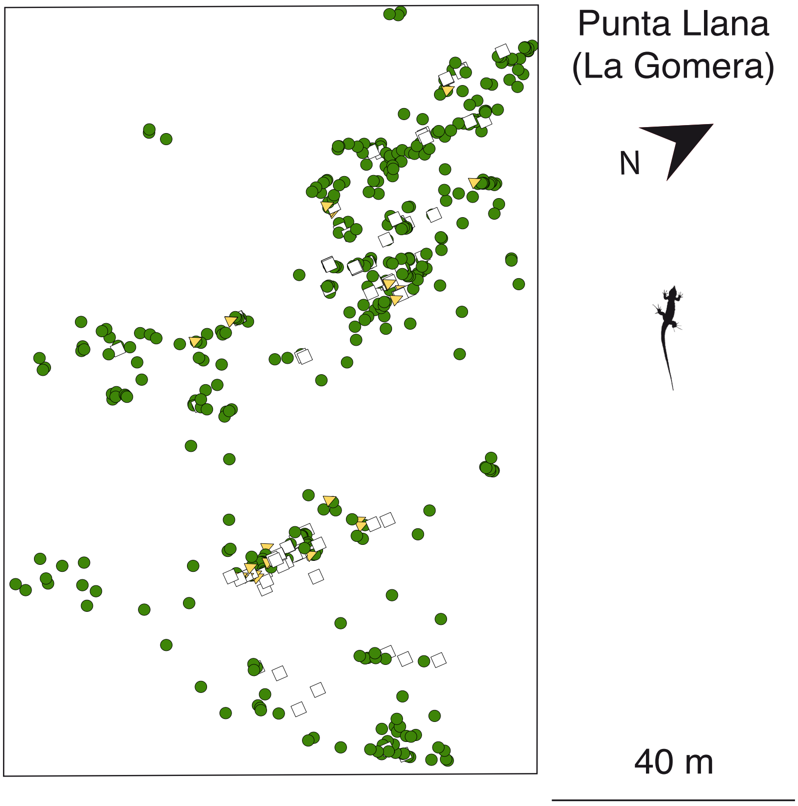
Punta Llana
(La Gomera)
N
40 m

Fig. S6 Spatial distribution of both adult individual plants of *Neochamaelea pulverulenta* (green circles) and seeds dispersed by lizards (red squares) at the three study 1-ha plots: Veneguera (Gran Canaria; *n*= 409 adults, 326 seeds), Teno (Tenerife; *n*= 778 adults, 305 seeds), and Punta Llana (La Gomera; *n*= 397 adults, 62 seeds). The adult plants sampled in the buffer area were also included as potential maternal plants for dispersed seeds sampled within the plot (*n*= 229 and 525 adults in Veneguera and Teno, respectively). The 1-ha plots are represented with continuous lines and the buffer limits with dashed lines. The three longest seed dispersal events in each plot are also indicated with lines (94.2 m, 72.7 m, 59.9 m, Veneguera; 46.4 m, 26.7 m, 22.4 m, Teno; 4.5 m, 2.2 m, 1.9 m, Punta Llana), arrows show the direction of the dispersal event. Lizard silhouettes are scaled to the maximum SVL.

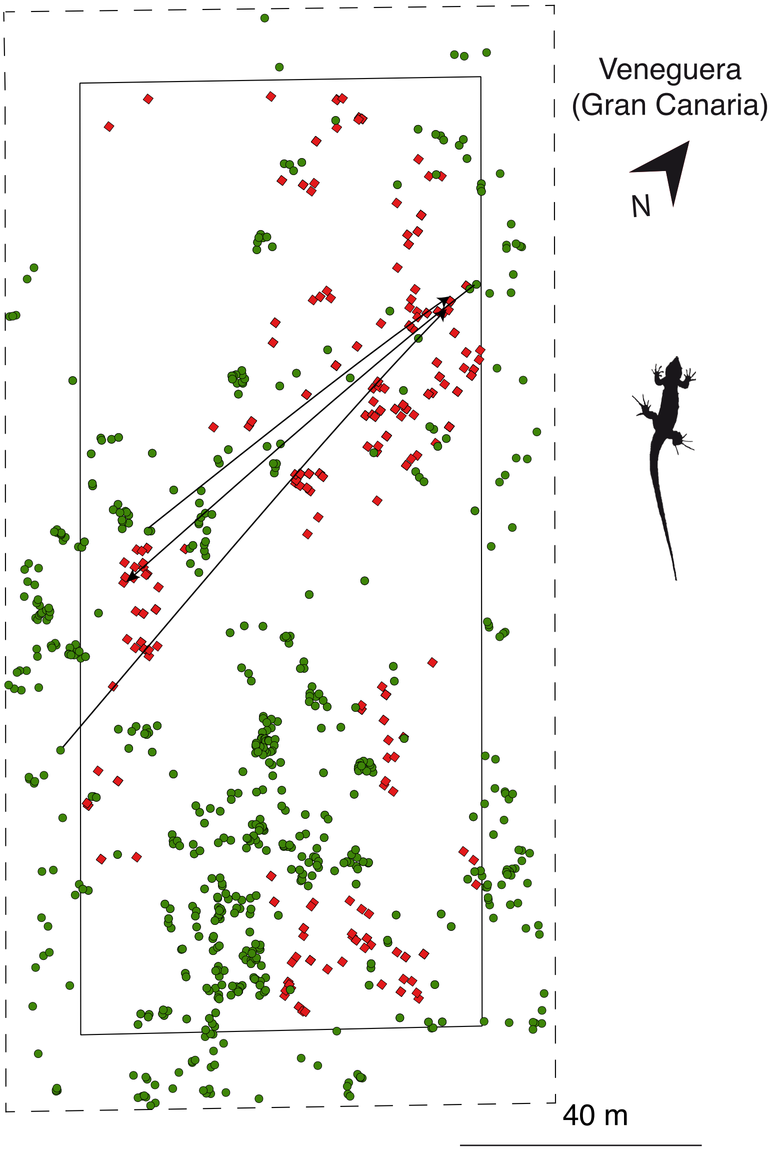

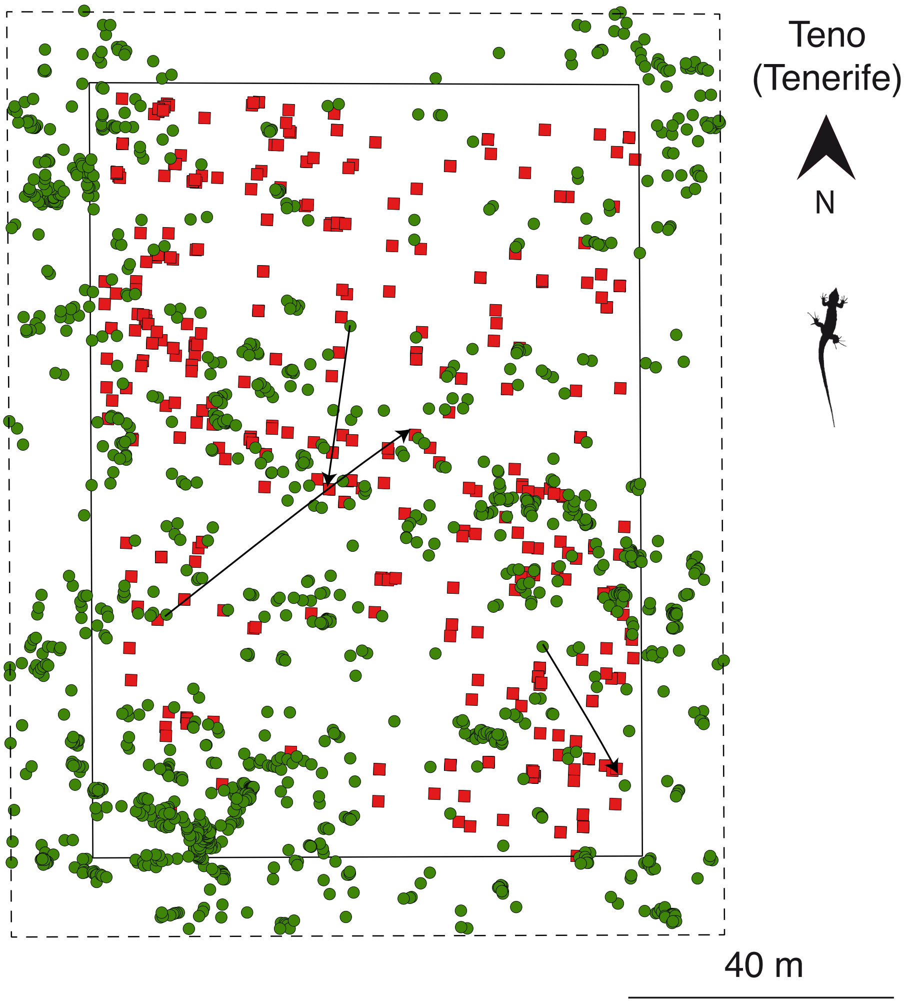

Teno
(Tenerife)
N
40 m

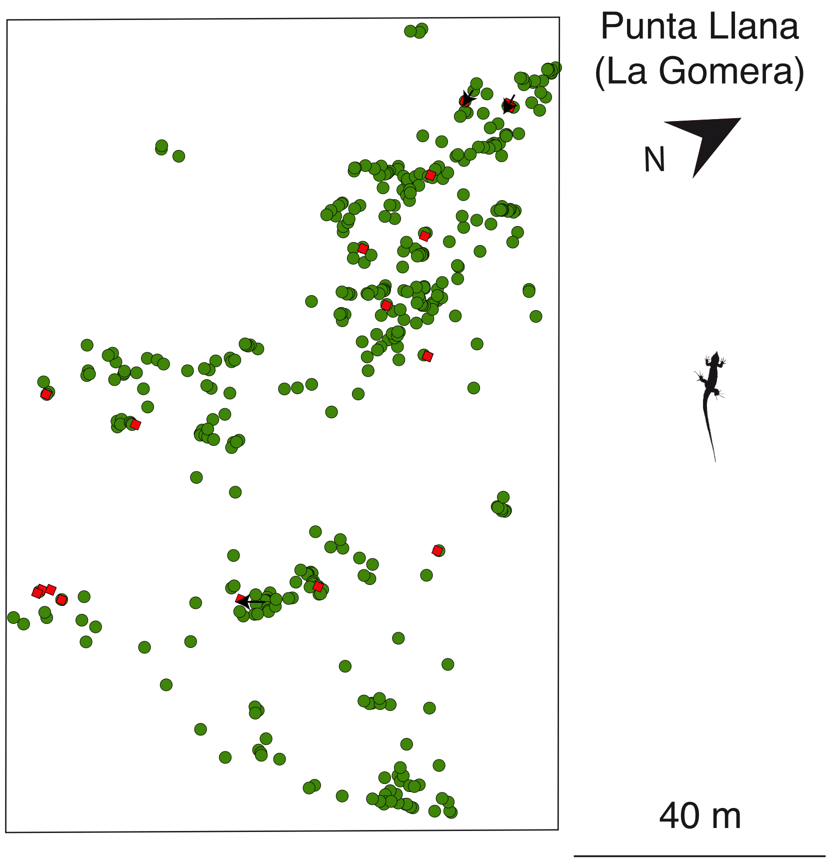
Punta Llana
(La Gomera)
N
40 m

Fig. S7 Output from ALLELEMATCH [46] at each 1-ha study plot: Veneguera (Gran Canaria), Teno (Tenerife), and Punta Llana (La Gomera) showing the optimal number of mismatches (black arrow) among multiple genotypes for robustly inferring unique individuals. Sampled genotypes matching below this threshold values were considered as unique genotypes. The curves represent the decay in the frequency of unique mismatches (dots) and the increase in the frequency of multiple matches (squares) between target seed endocarp and adult plant genotypes when increasing the number of mismatches.

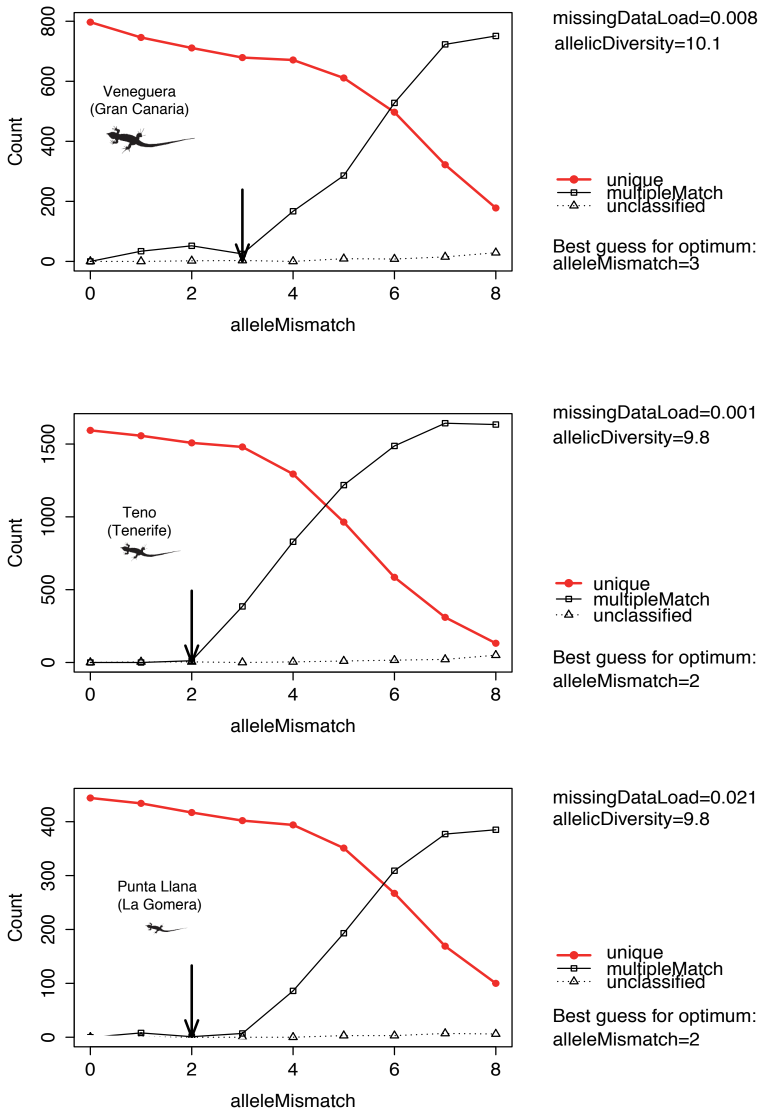

Table S1. Percentage of short-distance dispersal events (% SDD; percentage of seeds whose assigned mother plant was found within a 5-m radius) obtained in all studied populations. Percentages of SDD in the main study 1-ha plots (*) were determined by resampling (*n*= 999) a similar number of 5-m plots and seeds (*n*= 40) to those sampled in the replicated populations. Average values (± 1 SE) for each island are also indicated (see Fig. 1 for locations). Differences among islands in averaged % SDD are significantly different (*Chi-sq*= 8.02, $p < 0.05$; K-Wallis test).

Island	Locality	Coordinates	N plots	N seeds	% SDD
**Gran Canaria**			84	123	10.2 ± 5.9
	Barranco de Veneguera*	27°51'43" N<br>15°46'21" W	40	40	18.6 ± 5.7
	Barranco de Arguineguín 1	27°49°45'' N<br>15°40'46'' W	16	33	9.9
	Barranco de Arguineguín 2	27°48'13'' N<br>15°40'23'' W	8	15	6.7
	Barranco de Fataga	27°50'20'' N<br>15°34'10'' W	20	35	5.7
**Tenerife**			66	97	43.0 ± 6.5
	Teno Bajo*	28°21'13" N<br>16°54'33" W	40	40	41.9 ± 7.2
	Malpaís Punta de Rasca	28°00'42'' N<br>16°42'15'' W	16	35	37.1
	Caleta de Adeje	28°06'33'' N<br>16°44'26'' W	10	22	50.0

**La Gomera**			52	102	92.5 ± 6.5
	Punta Llana*	28º07'41" N<br>17º06'17" W	40	40	100 ± 0.0
	Taguluche	28º09’51’’ N<br>17º19’66’’ W	5	28	89.2
	Barranco del Medio	28º02’02’’ N<br>17º11’64’’W	7	34	88.2

Table S2. Summary of genetic diversity parameters obtained for different age classes of *Neochamaelea pulverulenta* across the 1-ha plots: Veneguera (Gran Canaria), Teno (Tenerife), and Punta Llana (La Gomera). *n* (adults; juveniles; saplings); Veneguera (409; 102; 164), Teno (778; 148; 61), and Punta Llana (397; 86; 26). Data are means ± 1 *SE*.

Population parameters		Veneguera (Gran Canaria)	Teno (Tenerife)	Punta Llana (La Gomera)
Expected Heterozygosity *(uHe)*	Adults	0.64 ± 0.06	0.62 ± 0.06	0.64 ± 0.06
	Juveniles	0.64 ± 0.05	0.62 ± 0.06	0.63 ± 0.07
	Saplings	0.61 ± 0.06	0.60 ± 0.06	0.62 ± 0.07
Observed Heterozigosity *(Ho)*	Adults	0.59 ± 0.06	0.60 ± 0.06	0.61 ± 0.06
	Juveniles	0.62 ± 0.06	0.57 ± 0.05	0.62 ± 0.07
	Saplings	0.58 ± 0.06	0.57 ± 0.06	0.61 ± 0.07
Effective number of alleles *(Ae)*	Adults	3.47 ± 0.49	3.42± 0.54	4.03 ± 0.79
	Juveniles	3.45 ± 0.45	3.38 ± 0.53	4.10 ± 0.77
	Saplings	3.17 ± 0.43	3.21 ± 0.44	3.48 ± 0.53
Allelic Richness *(AR)*	Adults	10.2 ± 1.60	10.2 ± 1.00	10.8 ± 1.40
	Juveniles	8.17 ± 1.19	7.75 ± 1.00	8.58 ± 1.23
	Saplings	8.67 ± 1.26	6.42 ± 0.64	6.83 ± 0.92
